# The Rate of Coronary Angiography Refusal in Older Patients with Non-ST Elevation Acute Coronary Syndrome and Its Impact on All-Cause Mortality

**DOI:** 10.14744/SEMB.2021.69908

**Published:** 2021-12-29

**Authors:** Kudret Keskin, Gokhan Cetinkal, Ozgur Selim Ser, Serhat Sigirci, Ahmet Gurdal, Kadriye Kilickesmez

**Affiliations:** Department of Cardiology, University of Health Sciences Turkey, Sisli Hamidiye Etfal Training and Research Hospital, Istanbul, Turkey

**Keywords:** Coronary artery disease, elderly, treatment refusal

## Abstract

**Objectives:**

Significant number older patients with acute coronary syndrome (ACS) cannot undergo coronary angiography (CAG) due to various comorbidities. Patient’s refusal of invasive procedures is common among old patients and has not been thoroughly investigated in the context of ACS. We wanted to assess CAG refusal rate and its impact on all-cause mortality in older patients with non-ST elevation acute myocardial infarction.

**Methods:**

In this retrospective study, patients over 75 years of age admitted with acute non-ST elevation ACS were included in the study. Patients were divided into three groups based on the treatment strategy; Group 1: Those who underwent CAG; Group 2: Refused; and Group 3: Deemed unsuitable for procedure due to severe comorbidities. The primary outcomes were to assess the patient refusal rate and its impact on all-cause mortality.

**Results:**

The study included 201 elderly patients. Eighty-two (41%) patients did not undergo CAG and of those, 48 (24%) had severe comorbidities, and 34 (17%) refused the procedure. The in-hospital mortality for patients who underwent, refused, or could not undergo CAG was 5.0%, 0%, and 16.7% (p<0.01); 30-day mortality 8.5%, 9.1%, and 25% (p=0.01); and long-term mortality was 20.2%, 35.3%, and 47.9% (p<0.01), respectively. The median follow-up was 12 months. Hazard ratio of treatment refusal for long-term mortality was 1.97 (1.02–3.87, 95% CI).

**Conclusion:**

Substantial number of elderly patients with ACS refuses to undergo CAG and this leads to increased mortality. Factors affecting patient behavior and the decision-making process should be explored.

The life expectancy of individuals continues to increase worldwide.^[1]^ One recent study estimated that by 2025, those over 80 years of age will comprise 6.4% of Europe’s population.^[2]^ As the population ages, so does the number older patients presenting with acute coronary syndrome (ACS) which is one of the most common cardiac disorder leading to mortality. Although current guidelines do not discriminate older patients from their younger counterparts in terms of treatment recommendations, invasive procedures particularly percutaneous coronary intervention (PCI) has unique problems mostly confined to advanced age.^[3,4]^ In addition to the procedural technical difficulties such as heavy calcification, tortuosity, and multivessel disease, there are certain accompanying comorbidities that often preclude invasive procedures particularly coronary angiography (CAG). Among such comorbidities frailty, renal failure and terminal malignancy are widely recognized and have been the basis of several studies.^[5]^ However, in daily practice we also witness that some patients refuse CAG for various reasons and this may be a relatively overlooked obstacle for implementation of appropriate therapy. For instance, in CRUSADE registry which evaluated patients with ST-elevation myocardial infarction, patient-related issues comprised 25% of cases who did not receive reperfusion therapy.^[6]^ Likewise, the rate of invasive strategy in patients with non-ST elevation ACS (NSTE-ACS) varies impressively from 32% to 95% among different nations and hospitals.^[7]^ Therefore, in our study, we aimed to investigate the rate of CAG refusal and its impact on all-cause mortality in elderly patients with NSTE-ACS and compare findings with patients who underwent CAG and with those whose CAG was not performed due to severe comorbidities.

## Methods

### Patients

In this retrospective cohort study, patients who were over 75 years of age admitted to the cardiology clinic from May 2015 to July 2017 with acute NSTE-ACS were included in the study. The diagnosis of NSTE-ACS was based on the presence of at least two of the following: Typical chest pain, dynamic electrocardiographic changes, and/or elevated troponin levels consistent with acute ischemia. Patients with STEMI and those who had end-stage malignancy with a life expectancy <12 months were excluded from the study. Ethical board approval was obtained from the local ethics committee (2003-05/06/2018). According to treatment strategy, patients were divided into three groups; Group 1: Patients with NSTE-ACS who underwent CAG; Group 2: Patients who refused to undergo CAG; and Group 3: Those whose CAG was not performed by the attending physician due to severe comorbidities.

Clinical and laboratory data were retrieved from hospital records. The clinical evaluation included age; sex; presence of hypertension, diabetes mellitus, accompanying comorbidities, and stroke; history of coronary revascularization, and in-hospital medications used. Laboratory data consisted of admission creatinine level, maximal creatinine level, estimated glomerular filtration rate, initial and maximum troponin I values, and other routine biochemical parameters. The diagnosis of acute kidney injury was determined using the Acute Kidney Injury Network criteria, in which at least 50% increase in the serum creatinine level was required for diagnosis.^[8]^

### Clinical Outcomes

The primary outcomes of the study were to assess the percentage of patients who refused CAG; and to evaluate in-hospital, 30-day and long-term all-cause mortality according to different treatment strategies. In-hospital mortality was evaluated through the review of hospital records, whereas 30-day and long-term mortality rates were identified through the national death notification system.

### Statistical Analysis

Distribution of data was assessed using the Kolmogorov–Smirnov test. The data for continuous variables are reported as mean±standard deviation or median and interquartile range according to the data distribution. Categorical variables are reported as numbers and percentages. Continuous variables were compared among the groups using one-way analysis of variance or the Kruskal–Wallis test. Event-free survival curves were generated using the Kaplan–Meier method. Differences in survival curves among the groups were assessed using the log-rank test. Cox regression analysis was used when calculating the hazard ratios for long-term mortality. A two-tailed p<0.05 was considered statistically significant. Statistical analysis was performed using SPSS 20 software (SPSS Inc., Chicago, IL).

## Results

**Figure 1.**
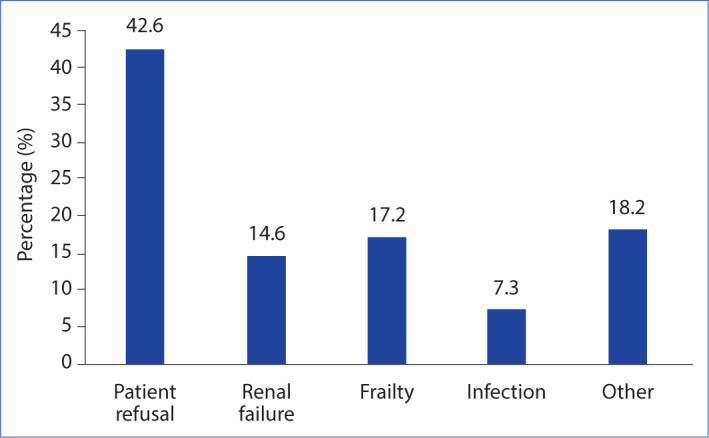
Patient refusal rates and common comorbidities observed in the older patients with non-ST elevation acute coronary syndrome.

We identified 201 elderly patients (aged 75 years and over) who had been hospitalized in our cardiology clinic because of NSTE-ACS. Baseline demographic and clinical characteristics are presented in Table 1. There were 112 (55.7%) female patients and mean age was 83±4 years. Overall 119 (59%) patients underwent CAG (Group 1) and remaining 82 (41%) were treated conservatively. Of these 82 patients, 34 (17%) refused the procedure (Group 2) and 48 (24%) could not undergo CAG because of severe medical conditions (Group 3). In group 3, the most common comorbidities were renal insufficiency (n=12, 14.6%), frailty (n=14, 17.1%), and in-hospital infection (n=6, 7.3%; Fig. 1).

**Table 1.** Baseline clinical characteristics, in-hospital medications, and laboratory values of elderly patients with NSTE-ACS

	**All patients (n=201)**	**(Group 1) Angiography Performed (n=119, 59%)**	**Angiography not performed**		**p**
			**(Group 2) Patient refusal (n=34, 17%)**	**(Group 3) Severe comorbidities (n=48, 24%)**	
Age (years)	83±4	81±4	84±4	85±5	<0.01
Gender (female) (n, %)	112 (55.7)	66 (55.5)	23 (67.6)	23 (47.9)	0.20
DM (n, %)	62 (30.8)	35 (29.8)	9 (26.5)	18 (37.5)	0.50
Hypertension (n, %)	151 (75.1)	92 (77.3)	25 (73.5)	34 (70.8)	0.66
Prior (n, %)										
Stroke	22 (10.9)	9 (7.6)	2 (6.1)	11 (22.9)	0.01
PCI	39 (19.4)	26 (21.8)	5 (14.7)	8 (17.0)	0.57
CABG	30 (14.9)	13 (10.9)	4 (11.8)	13 (27.1)	0.02
HF	35 (17.5)	12 (10.2)	7 (20.6)	16 (33.3)	<0.01
LVEF (%)	45 (35-55)	48 (40-55)	45 (35-52)	36 (30-60)	0.09
Revascularization rate (n, %)	68 (33)	68 (33)	-	-	N/A
Aortic stenosis (n, %)	14 (7.0)	6 (5.4)	4 (12.1)	4 (8.7)	0.39
CRF (eGFR<60%) (n, %)	105 (52.2)	56 (47.5)	19 (57.6)	30 (63.8)	0.13
Acute kidney injury (n, %)	30 (15.2)	20 (16.9)	4 (12.1)	6 (12.8)	0.69
Atrial Fibrillation (n, %)	33 (16.4)	17 (14.4)	8 (24.2)	8 (16.7)	0.40
Aspirin (n, %)	176 (88.0)	110 (92.4)	29 (87.9)	37 (77.1)	0.02
Clopidogrel (n, %)	161 (80.1)	99 (83.2)	27 (81.8)	35 (72.9)	0.31
Ticagrelor (n, %)	10 (5.0)	7 (5.9)	1 (3.0)	2 (4.2)	0.75
Inotropic therapy (n, %)	12 (6.0)	4 (3.7)	1 (2.9)	7 (14.6)	0.01
Laboratory values					
Glucose (mg/dl)	134 (110–183)	128 (106–171)	138 (110–173)	148 (122–215)	0.07
Leukocyte (×103)	8.9 (7.2–11.2)	8.5 (7.0–11.0)	8.7 ( 7.3–10.5)	10.4 (7.8–14.3)	0.04
Hemoglobin (gr/dL)	11.8 (10.6–13.0)	11.9 (10.8–13.0)	11.4 (10.0–12.5)	11.3 (10.1–13.0)	0.08
Platelet (×103)	220 (175–272)	222 (185–266)	212 (161–268)	228 ( 158–295)	0.56
Admission creatinine (mg/dL)	1.1 (0.8–1.4)	1.0 (0.8–1.3)	1.1 (0.7–1.8)	1.2 (1.0–1.6)	0.08
Maximum creatinine (mg/dL)	1.3 (1.0–1.8)	1.2 (0.9–1.6)	1.4 (0.8–1.9)	1.4 (1.1–2.0)	0.07
Admission Troponin (ng/ml)	0.8 (0.1–3.3)	0.7 (01.–2.9)	1.2 (0.1–4.8)	1.5 (0.3–3.4)	0.14
Maximum Troponin (ng/ml)	3.7 (0.7–12.1)	2.9 (0.5–12.3)	2.8 (1.1–13.6)	4.4 (0.9–11.1)	0.52
CRP (mg/L)	7.3 (2.9–32)	5.8 (2.9–26.5)	9.1 (2.3–37.7)	17.7 (5.8–67.8)	0.06
Total cholesterol (mg/dL)	164 (134–200)	178 (146–207)	154 (130–180)	154 (123–173)	<0.01
LDL cholesterol (mg/dL)	99 (70–123)	107 (76–134)	79 (61–116)	91 (68–107)	0.01
HDL cholesterol (mg/dL)	42 (32–51)	43 (32–52)	46 (38–50)	35 (27–46)	0.01
Triglyceride (mg/dL)	106 (82–143)	108 ( 90–155)	99 (67–119)		92 (73–126)	0.01
ALT (IU/L)	14 (10–22)	14 (10–20)	12 (10–23)	16 (11–29)	0.09
AST (IU/L)	22 (17–38)	20 (16–35)	26 (17–57)	25 (19–48)	0.02

DM: Diabetes mellitus; PCI: Percutaneous coronary intervention; CABG: Coronary artery bypass grafting; HF: Heart failure; LVEF: Left ventricular ejection fraction; CRF: Chronic renal failure; eGFR: Estimated glomerular filtration rate: NSTE-ACS: Non ST elevation acute coronary syndrome; CRP: C reactive protein; LDL: Low-density cholesterol; HDL: High-density cholesterol; ALT: Alanine transaminase; AST: Aspartate aminotransferase.

Patients who underwent CAG (Group 1) were relatively younger than the other two groups (81±4 vs. 84±4 and 85±5 years, respectively; p<0.01). Compared with the patients who underwent CAG, those who could not undergo CAG had statistically more episodes of prior stroke, coronary artery bypass surgery, and heart failure (p=0.01, p=0.02, and p<0.01, respectively). There was no statistical difference in terms of other baseline risk factors. Inotropic support was used more frequently in Group 3 compared to the other two groups. (Group 3 vs. Groups 1 and 2, p=0.01).

Laboratory values are presented in Table 1. There was no statistical difference among three groups with respect to the glucose, creatinine, hemoglobin, troponin I, and C-reactive protein values. However, the patients who could not undergo CAG (Group 3) had statistically lower total cholesterol, low-density lipoprotein cholesterol, and high-density cholesterol levels than Group 1.

### Clinical Outcomes

**Figure 2.**
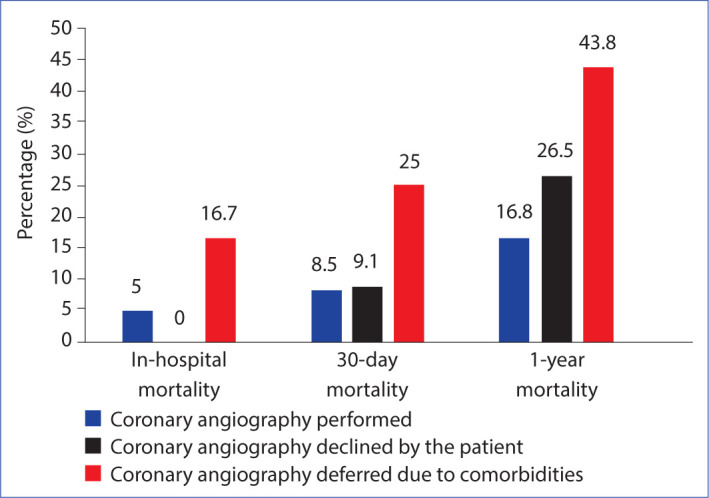
In-hospital, 30-day, and 1-year mortality rates according to the treatment strategies.

**Figure 3.**
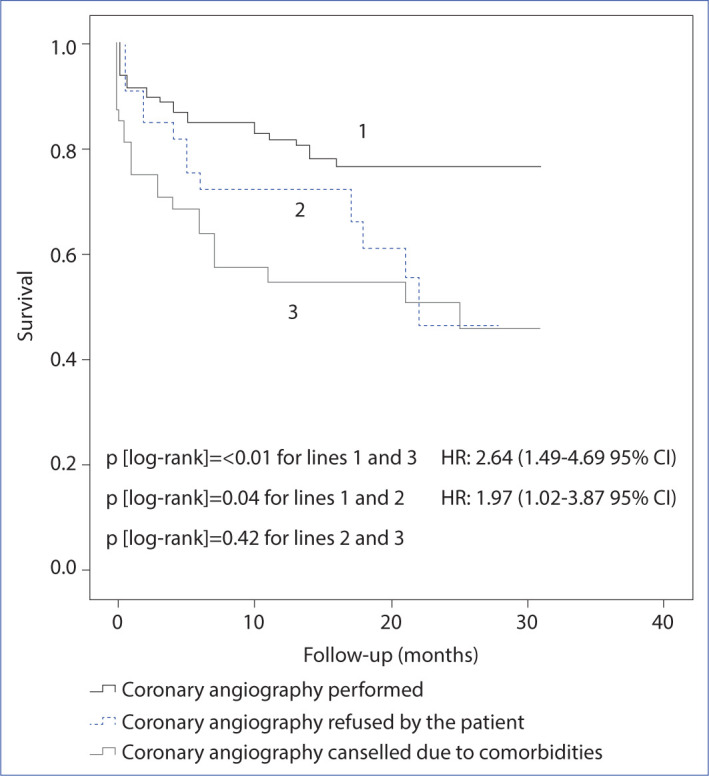
In-hospital, 30-day, and 1-year mortality rates according to the treatment strategies.

All-cause mortality rates are presented in Table 2. In hospital mortality for patients who underwent (Group 1), refused (Group 2), or could not undergo CAG (Group 3) were 5.0%, 0%, and 16.7% (p<0.01), respectively. Long-term mortality rate of these three groups was 20.2%, 35.3%, and 47.9% (p<0.01) (Fig. 2). Median follow-up was 12 months. Kaplan–Meier analysis showed that Group 1 had better survival rates than the two other groups (P [log-rank] = 0.04 and <0.01, respectively, Fig. 3). Hazard ratios for the long-term mortality of patients in Groups 2 and 3 based on Cox regression analysis were 1.97 (95% confidence interval [CI]: 1.02–3.87, p=0.04) and 2.64 (95% CI: 1.48–4.69, p<0.01), respectively.

**Table 2.** All-cause mortality rates

	**All patients (n=201)**	**(Group 1) Angiography Performed (n=119, 59%)**	**Angiography not performed**		**p**
			**(Group 2) Patient refusal (n=34, 17%)**	**(Group 3) Severe comorbidities (n=48, 24%)**	
In-hospital mortality	14 (7.0)	6 (5)	0 (0)	8 (16.7)	<0.01
30-day mortality	25 (12.6)	10 (8.5)	3 (9.1)	12 (25.0)	0.01
1-year mortality	50 (24.9)	20 (16.8)	9 (26.5)	21 (43.8)	<0.01
Long-term mortality	59 (29.4)	24 (20.2)	12 (35.3)	23 (47.9)	<0.01

## Discussion

In our study, we found that 41% of the study patients did not undergo CAG, and in 17% of cases, the reason was patient refusal. On the other hand, the most common comorbidities necessitating conservative management were renal failure, frailty, and in-hospital infection. Long-term survival analysis revealed that the worst outcome was seen in patients whose CAG could not be performed because of severe comorbidities, and the risk of all-cause mortality nearly doubled in patients who refused CAG compared to those who underwent CAG.

Although evidence supports invasive strategy even in elderly patients, it is also acknowledged that the implementation of guideline recommendations is not always possible in real-world practice.^[9]^ Studies that investigated the benefit of PCI in the elderly population mainly focused on the comorbidities as the main barrier against the implementation of an invasive strategy.^[10]^ Therefore, patient refusal remained a relatively overlooked etiology for deferring CAG. However, this problem may be a more significant factor than it seems and may lead to the under treatment of geriatric patients. For instance, according to the study led by Rothman et al. 16% of elderly patients tended to refuse one or more medical/surgical interventions, of which cardiac catheterization was the most common.^[11]^ Another study by Fried and Gillick reported that nearly 40% of older community dwelling patients refused medical treatments or testing during the 6 months before their death.^[12]^ Similarly, in a study conducted by Kashima et al. patient and family preferences comprised up to 40% of the reasons for refusing PCI among elderly patients with myocardial infarction.^[13]^ The most recent study from China reported that PCI was refused by almost one-quarter of the eligible patients with STEMI.^[14]^ In multivariate analysis, old age (more than 75 years), mild symptoms, and physician distrust were shown as important factors. A study from BLITZ-4 registry also addressed this issue and tried to establish a reasonable target for invasive strategy, where 85% was considered as the benchmark target for the treatment of consecutive patients with NSTE-ACS.^[15]^ In that study, patient refusal was the second common cause (21%) among reasons driving the choice for conservative strategy.

In our study, among patients in whom CAG was not performed, the patient refusal rate was 41%. Although there is limited data, we believe that patient refusal may be more prevalent in patients with NSTE-ACS compared to STEMI for several reasons. Atypical or even lack of symptoms, patient heterogeneity and delays to medical treatment are all factors more commonly seen in NSTEMI-ACS. Therefore, these features may have an impact on the patient’s perception of their disease severity, and thereby lead to refusal of CAG. Contrary to that, STEMI is a clinical condition in which patients are universally rushed to the catheterization laboratory and CAG is performed with almost no absolute contraindication. In these circumstances, even patient consent comes after the procedure. Thus, these two clinical myocardial infarction scenarios are completely different and patient refusal is not really an issue in STEMI.

Although our study did not investigate the reasons why patients refused the intervention, we believe that communication between the patient and health-care provider plays a pivotal role. It has been reported that older adults may have lower levels of health literacy, which may have a significant impact on treatment decisions.^[16]^ Patients can make fully informed and autonomous decisions to decline treatment. However, if CAG is declined because of distrusting the physician, poor communication, or inadequate information, these issues should definitely be addressed. Despite the emphasis on shared decision-making on critical issues, many older adults still accept the treatment recommendation offered by their physicians.^[17]^ Therefore, it is important that health-care providers play an active role during treatment decisions and obtaining informed consent.

On the other hand, there is not much evidence for the benefit of invasive strategy in patients who have severe comorbidities, as these patients are generally excluded from clinical trials.^[10]^ Renal failure, frailty, and in-hospital infection were the most common comorbidities for deferring the procedure in our study. This finding is in accordance with results reported in the literature, as these are the most common comorbid conditions. Renal failure, particularly acute deterioration accompanying chronic insufficiency, is common, and approximately 20% of elderly patients have chronic renal failure.^[18]^ Frailty, which was based on the attending physician’s discretion in our study, is seen in 26% of patients aged 80 or older, and further complicates the treatment strategy.^[19,20]^ Finally, as we have previously reported, the prevalence of acute infection was approximately 30% in octogenarians admitted because of ACS.^[21]^ Thus, infections in these patients should be evaluated rapidly, and appropriate therapy should be initiated in a timely manner.

### Limitations of the Study

First, this was a retrospective, single-center study. Second, the underlying reasons for patient refusal were not addressed. Third, frailty was based on the attending physician’s discretion; therefore, no frailty index was calculated. Finally, although we assessed all-cause mortality, we did not evaluate major adverse cardiac events separately.

## Conclusion

Although guidelines strictly recommend invasive strategy in older patients with NSTE-ACS, a substantial number still cannot undergo CAG because of severe comorbidities. In this context, patient-refusal may be an overlooked but important factor against the implementation of CAG. Since short- and long-term all-cause mortality rate of these patients were higher, factors affecting patient behavior with special emphasis on patient health-care provider communication should be explored to deliver the appropriate therapy.

### Disclosures

**Ethics Committee Approval:** Şişli Etfal Research and Training Hospital Ethics Committee (2003-05/06/2018).

**Peer-review:** Externally peer-reviewed.

**Conflict of Interest:** None declared.

**Authorship Contributions:** Concept – K.K., G.C., O.S.S. ; Design – K.K., G.C., O.S.S., S.S.; Supervision – K.K., A.G., K.K.; Materials – K.K., O.S.S., S.S.; Data collection &/or processing – K.K., G.C., O.S.S.; Analysis and/or interpretation – K.K., A.G., K.K.; Literature search – G.C., O.S.S., S.S.; Writing – K.K.; Critical review – K.K.
